# The Role of Gender in the Importance of Risk Factors for Coronary Artery Disease

**DOI:** 10.1155/2020/6527820

**Published:** 2020-07-29

**Authors:** Farshid Gheisari, Melika Emami, Hadi Raeisi Shahraki, Saeid Samipour, Parastoo Nematollahi

**Affiliations:** ^1^Ionizing and Non-Ionizing Radiation Protection Research Center, School of Paramedical Sciences, Shiraz University of Medical Sciences, Shiraz, Iran; ^2^Department of Epidemiology and Biostatistics, Faculty of Health, Shahrekord University of Medical Sciences, Shahrekord, Iran; ^3^Department of Chemical Engineering, School of Chemical and Petroleum Engineering, Shiraz University, Shiraz, Iran; ^4^School of Medicine, Shiraz University of Medical Sciences, Shiraz, Iran

## Abstract

Identification of risk factors and their importance in different genders is essential in order to prevent, diagnose, and manage coronary artery disease (CAD) properly. The present study aims to investigate the role of gender in the distribution of different risk factors in ischemic heart disease. This study is a cross-sectional study. More than one thousand (*N* = 1012) patients referring to the Nuclear Medicine Department in Namazi Hospital, Shiraz, Iran, from March 2017 to March 2018 were studied. The patients' demographic data and their clinical history were collected. The results of the myocardial perfusion scan were recorded and compared between groups. Statistical analysis was implemented by SPSS version 18.0, and *P* values below 0.05 were considered statistically significant. Out of the 1012 patients participating in this study, 698 (69%) were female and 314 (31%) were male. Ischemic heart disease (IHD) was significantly higher in men compared to women (19.1% versus 14.2%). The higher levels of systolic and diastolic blood pressures, along with older age, were a significant risk factor in women (*P* < 0.05). Previous myocardial infarction (MI), diabetes mellitus (DM), hypertension (HTN), and hyperlipidemia (HLP) had a strong correlation with IHD in our female population. Regarding the male subjects, previous MI and HLP had a lower correlation with IHD. Based on our logistic regression models, investigation of the simultaneous effects of risk factors on IHD showed that previous MI is the most effective risk factor in females (OR = 3.93) mostly in terms of residual ischemia in the infarcted myocardium. In the male population, on the other hand, HTN was identified as the most effective risk factor for IHD (OR = 2). In conclusion, we found that older age, higher blood pressure, DM, previous MI, HTN, and HLP have a significant association with IHD in the female population, whereas older age, DM, and HTN were significant risk factors for IHD in males. Also, the most effective factor for women was previous MI, while it was HTN for the male population.

## 1. Introduction

Atherosclerosis is characterized as an abnormal progressive remodeling process of the arterial wall in which various specified organs are endangered by ischemia, leading to coronary artery disease (CAD) and other related cardiovascular disorders [1–4]. Deaths from cardiovascular diseases are the most frequent cause of death globally, and the number of these deaths is expected to grow to 23.6 million in the following 20 years [2, 5, 6]. However, there are significant pharmacological and treatment protocol developments recently targeted at CVD patients, which improves their prognosis and could decrease the mortality rate [7].

Atherosclerosis pathophysiology could vary between genders [4, 8]. Additionally, coronary artery disease (CAD) is often underdiagnosed and viewed as a leading cause of female mortality. However, European data show that the degree of reduction in age-adjusted mortality for CVDs is more moderate for women than for men [9].

Several risk factors have been introduced for CAD, which are associated with acute MI in both women and men [10]. These include modifiable risk factors such as abnormal lipid levels, smoking, hypertension, abdominal obesity, lousy diet habit, mental stress, and diabetes [10]. There are also nonmodifiable risk factors, including male gender, age, and family history [10]. Because the initiation of clinical symptoms of CAD occurs later in women (about ten years later in women versus men), women diagnosed with CAD are often older with a likely higher prevalence of presented cardiovascular risk factors [2, 11].

Though traditional risk factors for CVD are expressed almost equally in both genders, determining the importance and relative weighting of these factors in each gender are not the same [4, 8].

According to a well-respected report, women and men had similar odds with respect to the relationship between acute myocardial infarction (AMI), smoking, elevated lipid levels, abdominal obesity, psychosocial influences, and vegetable and fruit intake [12]. Nonetheless, when it comes to the risk of hypertension and diabetes, as well as the protective effects of exercise and alcohol, the findings are more pronounced for women than for men [13–15]. Additionally, older women were reported to be more prone to hypertension than older men, which is strongly associated with stroke, left ventricular hypertrophy, and diastolic heart failure [13]. The cardiovascular problems associated with type 2 diabetes are more severe in women than in men, according to a meta-analysis of 37 prospective cohort studies [14]. Younger women suffer less from hypercholesterolemia compared to men, and although as they reach 65 and above, the mean LDL-cholesterol levels will increase in them [2, 15].

Different changes occurring during pregnancy and physiological differences in hormonal levels during menopause may explain this difference between men and women. Besides, women with hypercholesterolemia, reduced insulin sensitivity, volume excess during pregnancy, and also possible complications such as pre-eclampsia, gestational diabetes, and pregnancy-related hypertension are more prone to a higher risk of CVD later down the line [16]. Lastly, substantial changes in hormone levels during menopause processes (i.e., decreased levels of estrogen) may result in harmful alterations in cardiovascular risk factors [17]. Nonetheless, circulating estrogens control many metabolic pathways such as lipids, inflammatory markers, and the coagulation system [2, 18].

Identifying these risk factors and their gender-related disparities are, therefore, crucial for the proper prevention, treatment, and management of CAD. There are different diagnostic methods for diagnosing cardiovascular diseases such as electrocardiography, cardiac stress tests, myocardial perfusion scans, coronary computed tomography, and coronary angiography [19, 20]. As a more sensitive method for detecting myocardial ischemia, stress-rest myocardial perfusion imaging (MPI) was implemented in the mid-to-late 1970s [21]. The test was claimed to provide additional diagnostic and prognostic knowledge as opposed to ECG exercise and has since been significantly favored [22].

The radionuclide stress testing was considered a foundation for the practice and treatment of patients with suspected and confirmed coronary artery disease in the coming decades [23]. Given the global prevalence of CAD and its high mortality rate, it is essential to understand the significant risk factors for cardiovascular disease and to prevent and monitor these risk factors to reduce their adverse effects [8, 12, 16]. The appearance of one or more risk factors in an individual does not necessarily imply that heart disease is present, nor does it contribute to the absence of cardiovascular disease to regulate them [24]. Managing main risk factors can, moreover, help to reduce the risk of CAD and monitor the rate of disease progression or consequences and its complications [16, 20]. Considering the importance of CAD mortality and morbidity in both genders and the underestimation of heart disease risk in women and due to the lack of comprehensive information on risk factor differences in male and female, the present study aims to investigate the role of gender in each risk factor in cardiovascular disease.

## 2. Materials and Methods

This study is a cross-sectional study conducted in Namazi Hospital affiliated to the Shiraz University of Medical Sciences. More than one thousand (*N* = 1012) patients referring to the Nuclear Medicine Department in Namazi Hospital from March 2017 to March 2018 were studied. The inclusion criteria were patients needing IHD assessment and MPI and those aged older than 30 from both genders. The exclusion criteria were patients who had contraindications for undergoing stress-MPI based on clinical guidelines and had poor quality imaging, as well as patients unwilling to participate in the study. The patients' demographic data and their clinical history were collected by in-person interviews or reviewing their medical records. The data collection form included age, sex, weight, height, body mass index, laboratory results such as total cholesterol, triglyceride, FBS, LDL, and HDL, positive history of chronic disease (diabetes, hypertension, and hyperlipidemia), smoking, family history of ischemic heart disease (first-degree relative), past history of CCU admission, previous history of coronary angiography, prior myocardial infarction, and coronary artery bypass graft or percutaneous transluminal angioplasty. The results of the MPI test were recorded and compared between groups.

This study was found to be in accordance with the ethical principles and the national norms and standards for conducting medical research in Iran. The proposal was approved by the Research Ethics Committee of the Shiraz University of Medical Sciences (approval date: 2019-02-12; approval ID : IR.SUMS.MED.REC.1397.517).

### 2.1. Statistical Analysis

Statistical analysis was implemented by SPSS version 18.0, and *P* values less than 0.05 were considered statistically significant. The trend test was used to examine trends between the nominal and ordinal variables. In addition, the chi-square test and the independent *t* test were used where appropriate for assessing the association between the understudy factors and the status of IHD. To determine the simultaneous effect of potential risk factors on IHD, all variables with a *P* value 0.2 or less in univariate analysis were considered in multiple logistic regression (conditional backward elimination with alpha-to-remove = 0.1).

## 3. Results

Out of the 1012 patients participating in this study, 698 (69%) were female and 314 (31%) were male. The mean age of males and females was 57.4 (13.1) and 56.4 (12.0), respectively, which was not significantly different (*P*=0.23). The male and female participants of this study had also similar values in terms of mean blood pressure (93.3 versus 94.7, *P*=0.32), but the proportion of IHD among males was significantly higher than females (19.1% versus 14.2%, *P*=0.046).

The trend test showed a significant association between the proportion of IHD in females and a higher level of systolic or diastolic blood pressure (*P*=0.001 for systolic and *P*=0.009 for diastolic). Besides, female patients with IHD had a significantly higher blood pressure and were older compared to other females (*P* < 0.001 and *P*=0.002, respectively). On the other hand, there was no correlation between the proportion of IHD and the level of systolic and diastolic blood pressure in males (*P*=0.18 and 0.81, respectively). Also, males with IHD were significantly older than the other males (*P*=0.003), but they had similar values in terms of weight and blood pressure (Table 1).

As far as disease comorbidity was concerned, the proportion of IHD in females with previous MI was significantly higher than that in females without previous MI (44% versus 13.1%, *P* < 0.001), but there was no association between IHD and previous MI in males *P*=0.61. The chi-square test revealed that there was a significant association between IHD and DM in both genders (*P*=0.008 and 0.037 for females and males, respectively) as odds of IHD for DM patients were higher. HTN was another disease leading to increased odds of IHD in males and females (Table 2).

Although the proportion of IHD in male patients with and without HLP was the same, the proportion of IHD in female patients with HLP was significantly higher than that in females without HLP (17.2% versus 11.6%, *P*=0.03). Moreover, there was no association between smoking, opium addiction, and obesity with IHD in both males and females (Table 2).

In order to investigate the simultaneous effects of factors on the risk of IHD, two separate logistic regression models were fitted for males and females. We determined the final model using backward elimination with alpha-to-remove equal to 0.1.

The fitted model introduced previous MI as the most effective risk factor in females as odds of IHD in females with previous MI disease was 3.93 times more than that in other females. Our model also indicated that an eight-year increase in females' age would result in a 1.5-fold increase in their odds of having IHD (Table 3).

The regression model for males represented HTN as the most effective factor in IHD. Odds of IHD for males with HTN were about two times more than those for the other males (*P*=0.03). Moreover, increasing each 20 year in males' age would lead to a 1.5-fold increase in their odds of having IHD (Table 3).

## 4. Discussion

The role of the major cardiovascular risk factors in the development of IHD had unremarkable similarity in genders [8, 9]. As known, the overall risk factor level was more favorable in young females than males. However, the advantages of the female gender markedly diminished as they get older [4]. Consistent with our results, the statistical analysis showed that older age is a common risk factor in both genders.

Based on our study, IHD is more common in men (19.1% versus 14.2%). Moreover, in Wegner's study [25], the results showed that CAD and the first acute myocardial infarction occur several years later in women compared to men. Women's well-known biological defense against CAD before menopause can cause more than ten years of delay in presenting CAD symptoms in their bodies. Men under 55 are nearly four times more likely to develop MI than women. However, when they get older, the gender gap is decreased, but the prevalence of MI is still lower in women during their life as CHD levels do not increase suddenly in women at menopause. Finally, Wegner reported that overall 49% of men compared to 32% of women over 40 years of age are at a risk of CHD [25].

Likewise, Abbasi et al. [26] studied gender differences in terms of the risk of coronary artery disease (CAD) in Iran. They reported that of the 44,820 patients they studied, 37,358 had angiographically documented CAD. According to their study, CAD was more common in men (25,363) than women (11,995). They also found that the women were older, less educated, and more overweight than males [26].

In our study population, a higher level of systolic and diastolic blood pressure, along with older age, was a significant risk factor in women. We did not find a consistent trend between the level of blood pressure in males and the proportion of IHD; however, the logistic regression models showed that HTN was the most effective factor in IHD (OR = 3.54).

Wei et al. [27] performed a systematic review and meta-analysis to shed more light on sex-related variations regarding the impact of systolic blood pressure (SBP) on the risk of cardiovascular disease (CVD) and mortality. They estimated that the pooled effect size for increased risk of CVD per 10 mm Hg increase in SBP was 25 percent for women and 15 percent for men. For both women and men, the average increase in CVD mortality per 10 mm Hg SBP increase was the same. The results revealed that the risk of CVD per 10 mm Hg SBP rise for women was 1.1-fold higher than that for men, allowing some changes to age and baseline SBP. Nonetheless, the gender differences between men and women in CVD mortality were not relevant [27].

Studying the impact of disease comorbidity and ischemic heart disease revealed that previous MI, DM, HTN, and HLP have a strong correlation with IHD in our female population. However, in male subjects, previous MI and HLP had a lower association with IHD in our study population. In fact, residual ischemia in the infarcted myocardium is more common in women than men.

Madonna et al. [28] reviewed the impact of sex differences and diabetes on CAD and IHD. They reported that the cardiovascular risk in people with diabetes is two to three times higher than those without the disease, and it is believed that there is a difference in the risk of these diseases in women and men [28].

Eastwood and Doering also emphasized the fact that conventional risk factors differ among men and women [29]. Previously, it was believed that the gender difference was related to estrogen in the premenopausal period. However, they found that the most significant difference is diabetes mellitus, which leads to a notably higher increase in CAD risk in women compared to men, and the reason for this disparity in gender is unknown. Dyslipidemic women aged over 65 years are at a higher risk of CHD than men. Eastwood and Doering emphasized the effect of high triglyceride level and low levels of HDL on CAD risk in women [29]. Also, in their literature review, Oikonomu et al. [30] dealt with the gender difference in diabetes mellitus and CHD risk. They found that the mechanisms of insulin resistance and diabetes mellitus and CHD risk factor profiles are different in women. Still, the life-long variation of sex hormone levels in women complicates the research and understanding of this issue [30].

In conclusion, further studies are needed to illustrate the gender-specific hormone variation, risk factor profile, and gender difference in management outcomes based on diabetes mellitus and CVD interaction.

Previously, we studied the impact of smoking and opium abuse on IHD risk and found that there was no association between smoking, opium addiction, and obesity with IHD in both males and females.

Nonetheless, Abbasi et al. [26] reported that all the traditional risk factors were significantly dissimilar between both genders. Contrary to our findings, cigarette smoking was the most common risk factor in men (54%), whereas hypertension had the highest frequency (73%) in women with CAD. Comparing the risk factors between both genders showed that the frequency of male cigarette smokers or opium users was significantly higher than females; however, women had a higher frequency of a positive history for hypertension, diabetes mellitus, family history of CAD, hypertriglyceridemia, or hypercholesterolemia in their study population [26].

Based on our logistic regression models, investigation of the simultaneous effects of risk factors on IHD showed that previous MI is the most effective risk factor in females (OR = 3.93) mostly in terms of residual ischemia in infarcted areas. In the male population, HTN was realized as the most effective risk factor for IHD (OR = 2).

Our model also indicated that an eight-year increase in females' age would result in a 1.5-fold increase in their odds of having IHD and a 20-year increase in males had the same effect on IHD.

According to Blum and Blum [31], the prevalence of CAD was significantly lower in women <60 years than in older females. In women aged more than 60, the CAD rate elevated and exceeded the level of CAD risk reported in men. The atherosclerosis process in coronary arteries is different based on gender, and the difference would become larger at older ages (55 < years) [31].

Eastwood and Doering identified a high prevalence of CAD risk factors in US women aged between 20 and 70s. Hypertension, increased cholesterol level, smoking, overweight, and low physical activity were the most reported risk factors in the female population [29]. Conventionally, coronary heart disease (CHD) has been considered as a disease affecting mainly men, and for a long time, women were not studied for cardiovascular research purposes [32]. However, several recent studies highlighted the existence of a gender difference in terms of risk factors, symptoms, presentations, and applying the diagnostic and therapeutic procedures in women with IHD [8, 26, 29, 33]. Most risk factors contribute to IHD in both men and women, but the individual risk factors might exert their effects differently [8, 26, 29].

## 5. Conclusion

In conclusion, we found that older age, higher blood pressure, DM, previous MI, HTN, and HLP have a significant association with IHD in the female population; however, older age, DM, and HTN were significant risk factors for IHD in males. The most effective factor for women was previous MI; on the other hand, HTN was the most influential risk factor in the male population.

## Figures and Tables

**Table 1:** Risk factors for men and women and comparison of their association with IHD.

		Female		Male	
		With IHD	Without IHD	With IHD	Without IHD
Systolic [*N* (%)]	Normal	22 (9.6)	207 (90.4)	14 (12.5)	98 (87.5)
	Pre	33 (13.9)	205 (86.1)	30 (26.5)	83 (73.5)
	H1	30 (17.8)	139 (82.2)	8 (14.0)	49 (0.86)
	H2	11 (20.0)	44 (80.0)	6 (22.2)	21 (77.8)
	H3	3 (42.9)	4 (57.1)	2 (40.0)	3 (60.0)
Trend test *P* value		0.001		0.19	
Diastolic [*N* (%)]	Normal	55 (11.7)	414 (88.3)	38 (17.9)	174 (82.1)
	Pre	8 (21.1)	30 (78.9)	9 (36.0)	16 (64.0)
	H1	25 (17.4)	119 (82.6)	12 (19.7)	49 (80.3)
	H2	11 (23.9)	35 (76.1)	1 (7.1)	13 (92.9)
	H3	0 (0)	1 (100)	0 (0)	2 (100)
Trend test *P* value		0.009		0.81	
Age [mean (STD)]		63.0 (10.7)	55.3 (11.9)	61.3 (10.1)	56.5 (13.6)
*t*-test *P* value		<0.001		0.003	
Weight [mean (STD)]		69.3 (13.8)	68.3 (12.7)	74.1 (11.4)	73.4 (12.8)
*t*-test *P* value		0.48		0.70	
Blood pressure [mean (STD)]		104.1 (46.4)	93.1 (14.4)	96.0 (15.2)	92.7 (15.6)
*t*-test *P* value		0.02		0.14	

All the information is presented as frequency (%).

**Table 2:** Disease comorbidity and conditions in the study populations and their role in IHD.

Comorbidity disease		Female		Male	
		With IHD	Without IHD	With IHD	Without IHD
Previous MI [N (%)]	No	88 (13.1)	585 (86.9)	52 (18.7)	226 (81.3)
	Yes	11 (44.0)	14 (56.0)	8 (22.2)	28 (77.8)
Chi-square test *P* value		<0.001		0.61	
DM [*N* (%)]	No	57 (11.8)	425 (88.2)	41 (17.9)	188 (82.1)
	Yes	42 (19.4)	174 (80.6)	19 (22.4)	66 (77.6)
Chi-square test *P* value		0.008		0.037	
HLP [*N* (%)]	No	44 (11.6)	335 (88.4)	41 (19.0)	175 (81.0)
	Yes	55 (17.2)	264 (82.8)	19 (19.4)	79 (80.6)
Chi-square test *P* value		0.03		0.93	
HTN [*N* (%)]	No	25 (8.3)	276 (91.7)	22 (13.3)	143 (86.7)
	Yes	74 (18.6)	323 (81.4)	38 (25.5)	111 (74.5)
Chi-square test *P* value		<0.001		0.006	
Smoking [*N* (%)]	No	79 (14.3)	473 (85.7)	34 (19.1)	144 (80.9)
	Yes	20 (13.7)	126 (86.3)	26 (19.1)	110 (80.9)
Chi-square test *P* value		0.85		0.99	
Opium [*N* (%)]	No	84 (15.1)	473 (84.9)	37 (20.1)	147 (79.9)
	Yes	15 (10.6)	126 (89.4)	23 (17.7)	107 (82.3)
Chi-square test *P* value		0.18		0.59	
Obesity [*N* (%)]	No	59 (13.8)	368 (86.2)	50 (19.8)	203 (80.2)
	Yes	40 (14.8)	231 (85.2)	10 (16.4)	51 (83.6)
Chi-square test *P* value		0.73		0.55	

All the information is presented as frequency (%).

**Table 3:** Results of separate regression models for women and men.

	Female		Male	
	OR (95% CI)	*P* value	OR (95% CI)	*P* value
Age	1.05 (1.03,1.07)	<0.001	1.02 (1.0,1.05)	0.04
HTN status (yes vs no)	—	—	1.96 (1.08,3.54)	0.03
Blood pressure	1.02 (1.0,1.04)	0.005	—	—
Previous MI (yes vs no)	3.93 (1.59,9.73)	0.003	—	—
Opium consumption status (yes vs no)	0.56 (0.3,1.06)	0.07	—	—

## Data Availability

The datasets used and analyzed during the current study are available from the corresponding author on reasonable request.
